# ToxiVerse: chemical bioprofiling, toxicity data sharing and customizable predictive modeling

**DOI:** 10.1093/bioinformatics/btag617

**Published:** 2026-08-22

**Authors:** Prasannavenkatesh Durai, Daniel P Russo, Yitao Shen, Tong Wang, Elena Chung, Lang Li, Hao Zhu

**Affiliations:** Center for Biomedical Informatics and Genomics, Tulane University School of Medicine, New Orleans, LA 70112, United States; Department of Chemistry and Biochemistry, Rowan University, Glassboro, NJ 08028, United States; Center for Biomedical Informatics and Genomics, Tulane University School of Medicine, New Orleans, LA 70112, United States; Department of Chemistry and Biochemistry, Rowan University, Glassboro, NJ 08028, United States; Center for Biomedical Informatics and Genomics, Tulane University School of Medicine, New Orleans, LA 70112, United States; Department of Chemistry and Biochemistry, Rowan University, Glassboro, NJ 08028, United States; Center for Biomedical Informatics and Genomics, Tulane University School of Medicine, New Orleans, LA 70112, United States; Department of Chemistry and Biochemistry, Rowan University, Glassboro, NJ 08028, United States; Department of Biomedical Informatics, College of Medicine, The Ohio State University, Columbus, OH 43210, United States; Center for Biomedical Informatics and Genomics, Tulane University School of Medicine, New Orleans, LA 70112, United States; Department of Chemistry and Biochemistry, Rowan University, Glassboro, NJ 08028, United States

## Abstract

**Motivation:**

Chemical toxicity assessment is critical for drug development and environmental safety. Computational models have emerged as a promising alternative to animal testing and now play a significant role in efficiently evaluating new chemicals. To address the urgent need for user-friendly machine learning tools in computational toxicology, we developed ToxiVerse, a public web-based platform.

**Results:**

ToxiVerse provides automatic chemical bioprofiling, curated toxicity datasets, and a predictive modeling interface designed for researchers who lack programming expertise. The platform comprises three integrated modules: (i) Bioprofiler, which provides chemical descriptors by combining chemical-bioactivity data from PubChem assays with a machine learning-based data gap-filling procedure; (ii) Database, which hosts ∼50 000 curated chemicals covering diverse toxicity endpoints; and (iii) Cheminformatics, which enables dataset upload, chemical curation, and automatic generation of quantitative structure–activity relationship models for toxicity prediction.

**Availability:**

The tool is accessible at www.toxiverse.com, and source code is available at https://github.com/zhu-research-group/toxiverse.

## Graphical abstract

**Graphical Abstract:**
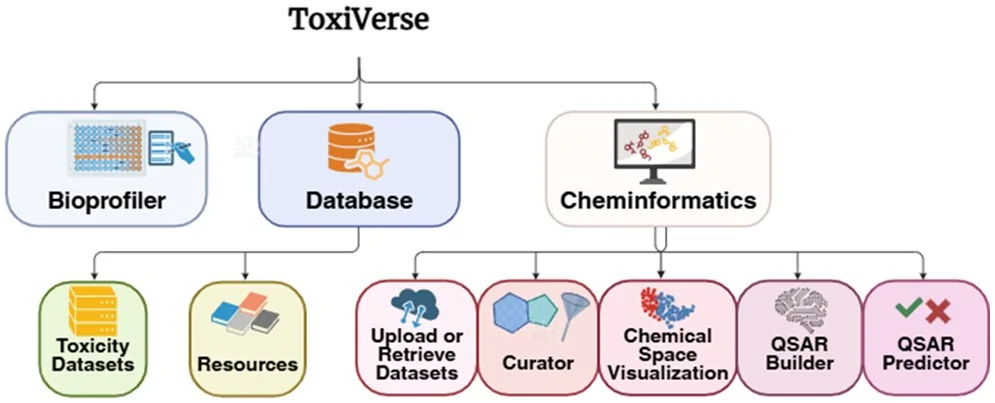

## 1 Introduction

Chemical safety evaluation is fundamental to safeguarding both human and environmental health and underpins decisions made by regulatory agencies (Guo *et al.* 2023). In drug development, drug-induced side effects and toxicities are major contributors to both early- and late-stage failures, responsible for up to 40% of preclinical attritions reported in 2015 and about 20% of Phase II and Phase III attritions reported in 2008 (Muster *et al.* 2008, Waring *et al.* 2015). Chemical toxicity also imposes a major public health burden, with more than two million adverse drug events and approximately 1 00 000 deaths reported in the United States in 1994 alone, linked to drug-induced toxicity (Muster *et al.* 2008). Current regulatory frameworks still rely heavily on animal testing, which is time-consuming, costly, and ethically controversial (Gibb 2008, Ford 2016, Pognan *et al.* 2023). These limitations have catalyzed a shift toward alternative approaches, including *in vitro* testing and *in silico* modeling (Ford 2016). The progress of computational toxicology along with this shift has been driven by advances in computational power and new algorithm development, as well as the increasing availability of large public databases of toxicity data (Shen and Nicolaou 2019, Jia *et al.* 2023, Wang *et al.* 2024). For instance, Tox21 (Richard *et al.* 2021) has screened more than 10 000 compounds across diverse *in vitro* assays to support high-throughput, mechanism-informed toxicological profiling. The resulting toxicity data are publicly available through the Tripod database (https://tripod.nih.gov/tox/) and http://www.ncbi.nlm.nih.gov/pcassay? term=tox21 (Richard *et al.* 2021).

Building on the increasing availability of data and new algorithms, various computational tools have been developed to meet the growing demand for new chemical toxicity evaluations (Seal *et al.* 2025). These include online platforms such as ADMETlab 3.0 (Fu *et al.* 2024), admetSAR 3.0 (Gu *et al.* 2024), ProTox 3.0 (Banerjee *et al.* 2024), variable nearest neighbor (vNN)-ADMET (Schyman *et al.* 2017), EMolTox (Ji *et al.* 2018), and VenomPred 2.0 (Di Stefano *et al.* 2024), as well as tools for specific toxicities like Pred-hERG 5.0 (Braga *et al.* 2015), PredSkin 3.0 (Borba *et al.* 2021), and CardPred (Lee *et al.* 2019). For example, ADMETlab 3.0 is a web-based platform that can predict around 80 endpoints related to Absorption, Distribution, Metabolism, Excretion, and Toxicity (ADMET) using Directed Message Passing Neural Network (DMPNN) models (Fu *et al.* 2024). The admetSAR 3.0 tool supports predictions for over 100 ADMET endpoints and combines classification and regression models built with a contrastive learning-based multi-task graph neural network framework (CLMGraph) (Gu *et al.* 2024). ProTox 3.0 provides chemical structures, protein targets, and pathway information as outputs and predicts 28 newly added toxicity endpoints using models developed with deep neural network (DNN) or Random Forest (RF) methods (Banerjee *et al.* 2024). The vNN method is a similarity-based Quantitative Structure-Activity Relationship (QSAR) approach used to predict ADMET properties (Schyman *et al.* 2017). Other toolboxes like QsarDB (Ruusmann *et al.* 2015) offer a digital repository of validated QSAR models, while VEGA-QSAR (https://ceur-ws.org/Vol-1107/paper8.pdf) and the OECD QSAR Toolbox provide built-in models and read-across tools for predicting chemical toxicities (Schultz *et al.* 2018).

Recent improvements in these chemical toxicity evaluation tools include integrating deep learning methods (e.g. DNN, DMPNN, and CLMGraph) into model developments, which enhance model performance and broaden endpoint coverage (Wang *et al.* 2024). However, model interpretability continues to be a challenge, particularly in deep learning-based models due to their black-box nature (Jia *et al.* 2023, Seal *et al.* 2025). Training data size and quality also impacts model predictivity. Many publicly available toxicity databases suffer from issues such as inconsistent annotations, missing metadata, lack of rigorous curation, non-standardized data formats, and species discrepancies (Wu *et al.* 2023, Wang *et al.* 2024). These problems can introduce significant bias into toxicity modeling results. Few existing chemical toxicity evaluation tools offer essential features such as batch processing, support for custom dataset modeling, and standardized modeling reports (Seal *et al.* 2025). Moreover, most existing computational toxicity tools require programming expertise, making them less accessible for researchers without such backgrounds (Wang *et al.* 2024, Seal *et al.* 2025). Although web-based platforms are popular, many rely only on pre-trained models and offer limited flexibility for user data input and customized predictions (Yang *et al.* 2018, Wang *et al.* 2024, Seal *et al.* 2025).

In this study, we present ToxiVerse, an online chemical toxicity modeling platform designed to address these challenges by delivering a user-friendly web-based toolbox that integrates automated chemical bioprofiling based on PubChem assay data, curated toxicity datasets across diverse endpoints, and customizable machine learning (ML) modeling. ToxiVerse has three application modules. First, the Bioprofiler module that profiles chemicals against their bioassay outcomes from PubChem (Low *et al.* 2013, Zhu *et al*. 2016, Russo *et al.* 2017, Ciallella *et al.* 2022a) and predicts outcomes for target chemicals with inconclusive or missing data, thereby generating comprehensive chemical descriptors. Second, the Database module provides access to rigorously curated chemical toxicity datasets covering around 50 000 chemicals, mostly with data on critical endpoints such as hepatotoxicity, carcinogenicity, and developmental toxicity. Third, the Cheminformatics module offers utilities for dataset upload and retrieval from the PubChem database, dataset curation, and automated QSAR model generation using widely adopted chemical descriptors and ML algorithms. Users can build models using any provided ToxiVerse datasets or their own data and select the most suitable models to predict their chemicals of interest. By providing a user-friendly ML platform, ToxiVerse empowers researchers worldwide to perform interpretable, scalable, and computational toxicity modeling for chemical risk assessment purposes.

## 2 Methods

### 2.1 Platform implementation

ToxiVerse platform was developed as a modular web application using the Flask framework (Flask 2.2, Python 3.11) and deployed within a Docker environment. The front-end interface was implemented with HTML,CSS, JavaScript and jQuery 3.4.1, styled using Bootstrap 4.1.0. Interactive visualizations are implemented with Plotly 5.9.0, while server-side plots are rendered using Matplotlib 3.7.0 and Seaborn 0.13.0. Server-side execution is handled by Gunicorn, while Redis 4.4.0 is employed as the backend for asynchronous task queuing via Redis Queue. Curated datasets and PubChem chemical-bioactivity data are stored in an SQLite database, accessed through Flask-SQLAlchemy 3.0.2. A RESTful API supports core functionalities, including Principal Component Analysis (PCA) visualization, chemical data distribution plotting, access to relevant bioassay data of endpoints, and dataset downloads. Data curation and descriptor calculation were performed using RDKit 2022.09.2 (www.rdkit.org), while predictive modeling was carried out with scikit-learn (1.2.0). The web server is publicly accessible without requiring user login.

### 2.2 Bioprofiler module

#### 2.2.1 Database construction with PubChem data

A SQLite database leveraging PubChem data was built using two primary datasets downloaded from the PubChem FTP (Kim *et al.* 2025): a bioactivities set and a compound-to-InChIKey mapping set. The bioactivities set contains experimental assay results, including PubChem compound identifiers (CIDs), PubChem assay identifiers (AIDs), and reported chemical activity outcomes (*active*, *probe*, *inactive*, *inconclusive, or unspecified*). The mapping set provides standardized chemical structures in the form of InChI strings for each CID. These two datasets were merged based on CID, and records with missing or incomplete InChI strings were removed to ensure consistent chemical representation. The final database used for profiling in this study contains four key fields: CID, AID, Activity Outcome, and InChI string.

#### 2.2.2 Assay selection and machine learning model development

Mutual information (MI) scores were calculated using scikit-learn’s classification module to guide assay selection. MI measures how informative assay outcomes are about the overall activity of chemicals. First, the bioactivity data collected from PubChem assays for target chemicals were transformed into a chemical-bioactivity matrix (i.e. initial bioprofile), where active values were encoded as 1 and inactive or inconclusive values were encoded as 0. Next, each chemical was assigned a binary overall activity label, defined as active if it was active in at least one assay. Finally, the MI between the activity outcome of each assay and the overall activity label was calculated. An assay achieves a high MI score when both its actives (1) and inactives (0) closely match the overall activity labels of as many chemicals as possible. The more consistently an assay distinguishes overall active from inactive chemicals, the higher its MI is.

For chemicals from the selected assays, ML models were developed to fill data gaps for compounds lacking experimental *active*/*inactive* confirmation. To this end, RF classifiers were trained using Extended-connectivity fingerprints (ECFP) 6 with a radius of 3 and a bit length of 2048, and their performance was evaluated using commonly adopted binary classification metrics. RF is an ensemble learning algorithm that constructs many individual decision trees during training and aggregates their outputs to generate predictions (Breiman 2001). ECFP are circular topological fingerprints that encode local atomic neighborhoods into fixed-length binary vectors (Rogers and Hahn 2010).

### 2.3 Database module

Curated chemical datasets were collected from multiple resources, most of which have been described in our previous studies (Klopman *et al.* 2000, Moda *et al.* 2007, Zhu *et al.* 2009, Sedykh *et al.* 2013, Kim *et al.* 2014, Wang *et al.* 2015, Mulliner *et al.* 2016, Zhao *et al.* 2017, Ciallella *et al.* 2021, Ciallella *et al.* 2022b, Aljarf *et al.* 2023, Béquignon *et al.* 2023, Chung *et al.* 2023, Knox *et al.* 2024, Zdrazil *et al.* 2024, Daood *et al.* 2025). Duplicate chemicals with conflicting labels were handled according to endpoint type. For binary endpoints, when the same chemical had different labels across sources, the final label was assigned by majority vote across the available labels. For continuous endpoints, duplicate numeric values from comparable sources were consolidated by calculating the mean value. These datasets were used to construct an SQLite database, where each chemical was assigned a unique ToxiVerse-ID, canonical SMILES were generated and stored, and PubChem CID was included when available. Each dataset can be downloaded and explored through a histogram showing the distribution of chemical counts across activity value ranges.

In addition, relevant bioassays for each endpoint across all datasets were extracted using bioactivity data collected from PubChem with the Bioprofiler module. Based on the bioprofile data of chemicals in each dataset, bioassays relevant to the corresponding endpoint were identified. First, the counts of *active*, *inactive*, and *inconclusive* compounds were computed for each bioassay in the bioprofile data. The active rate for bioassay *i* was calculated as:


(1) $$\text{Active}\;{\text{Rate}}_{i}=\frac{{\text{Active}}_{i}}{{\text{Active}}_{i}+{\text{Inactive}}_{i}}$$

To harmonize active rates while accounting for the active rates of other assays in the bioprofile, Bayesian smoothing was applied to adjust the active rates calculated above. The average active rate μ across all relevant bioassays was computed as:


(2) $$\mu =\frac{\sum_{i=1}^{N}{\text{Active}}_{i}}{\sum_{i=1}^{N}\left({\text{Active}}_{i}+{\text{Inactive}}_{i}\right)}$$

where *N* denotes the total number of bioassays in the bioprofile. For each bioassay *i*, the Bayesian Adjusted Score was then calculated as:


(3) $$\text{Adjusted}\;{\text{Score}}_{i}=\frac{{\text{Active}}_{i}+k\cdot \mu }{{\text{Active}}_{i}+{\text{Inactive}}_{i}+k}$$

where *k *= 100 is the Bayesian smoothing parameter that balances the influence of the average activity rate with assay-specific observations.

To reduce the influence of insignificant bioassays, assays with fewer than 500 total observations were excluded before ranking. The Bayesian ranking procedure was repeated independently for each dataset endpoint using *k* values of 25, 50, 100, 200, and 500. Across the dataset-endpoint files, the top 500 assay lists were highly stable across moderate *k* values, with median overlaps of 99.8% between *k* = 50 and *k* = 100, and 99.6% between *k* = 100 and *k* = 200. Therefore, *k* = 100 was retained as a balanced smoothing parameter. For each dataset endpoint, the 500 related bioassays with the highest Adjusted Scores after small-sample filtering were selected.

### 2.4 Cheminformatics module

#### 2.4.1 Dataset and chemical curator

Input chemical files, in either CSV or SDF format, can be uploaded by users through the Cheminformatics module. Users can also import datasets directly from PubChem by providing the relevant AID as input. The curator function leverages scripts from the ChEMBL Structure Pipeline (Bento *et al.* 2020), an open-source cheminformatics framework designed to systematically clean, standardize, and validate the input chemical structures. Built on top of RDKit, the pipeline performs rigorous chemical structure curation to ensure chemical integrity and consistency. This procedure detects invalid or ambiguous stereochemistry, problematic valence states, illegal bond types, and undesirable substructures (e.g. salts, solvents, metals). Then the standardization step includes kekulization, hydrogen removal, SMIRKS-based normalization, uncharging, and conformer cleanup.

#### 2.4.2 Chemical space visualization

The PCA dimensionality reduction method was employed to visualize the chemical space of the datasets. Descriptors are calculated using RDKit and then standardized. Dimensionality reduction is performed with scikit-learn to extract the top three principal components. The resulting PCA coordinates, together with CIDs and activity labels, are used to generate a 3D scatter plot. Compounds are color-coded by activity classification, enabling users to explore chemical clusters within each dataset.

#### 2.4.3 QSAR modeling

Based on user selection, molecular descriptors or fingerprints (RDKit molecular descriptors, ECFP6 fingerprints, and Functional-Class Fingerprint (FCFP) 6) are calculated for each chemical in the target dataset and normalized if necessary. FCFP are feature-based circular fingerprints that encode local pharmacophoric environments into fixed-length binary vectors (Rogers and Hahn 2010). In this function, FCFP6 fingerprints are generated using a bond radius of three, resulting in 2048-bit vectors that capture feature-level substructural patterns up to six bonds in diameter. The module supports classification or regression modeling using ML algorithms, including RF, Support Vector Machines (SVM), and *k*-Nearest Neighbors (*k*-NN). Model hyperparameters are optimized via grid search with five-fold cross-validation. For classification, a default probability threshold of 0.5 is used for balanced endpoints to classify chemicals (1 = active/toxic, 0 = inactive/non-toxic). Class ratios worse than 1:2 are treated as imbalanced. For imbalanced endpoints, the classification threshold is automatically estimated from the cross-validated out-of-fold predicted probabilities using Youden’s J statistic, which balances sensitivity and specificity. The best-performing model by cross-validation metrics can be saved for future predictions. SVM is a supervised learning algorithm that identify the optimal separating hyperplane between classes in a high-dimensional feature space (Cortes and Vapnik 1995). The *k*-NN is a distance-based, non-parametric algorithm that classifies a query compound according to the majority class of its *k* most similar compounds in the training set (Cover and Hart 1967).

#### 2.4.4 QSAR prediction

By selecting a generated model, chemicals to be predicted can be input through two options: (i) file upload as a CSV or SDF file, or (ii) direct entry of SMILES strings. For CSV files, the system automatically detects a column named “SMILES” or allows users to manually specify the column containing the SMILES strings of the chemicals. The predicted results are provided as CSV files.

## 3 Results and discussion

### 3.1 Overview of ToxiVerse

**Figure 1:**
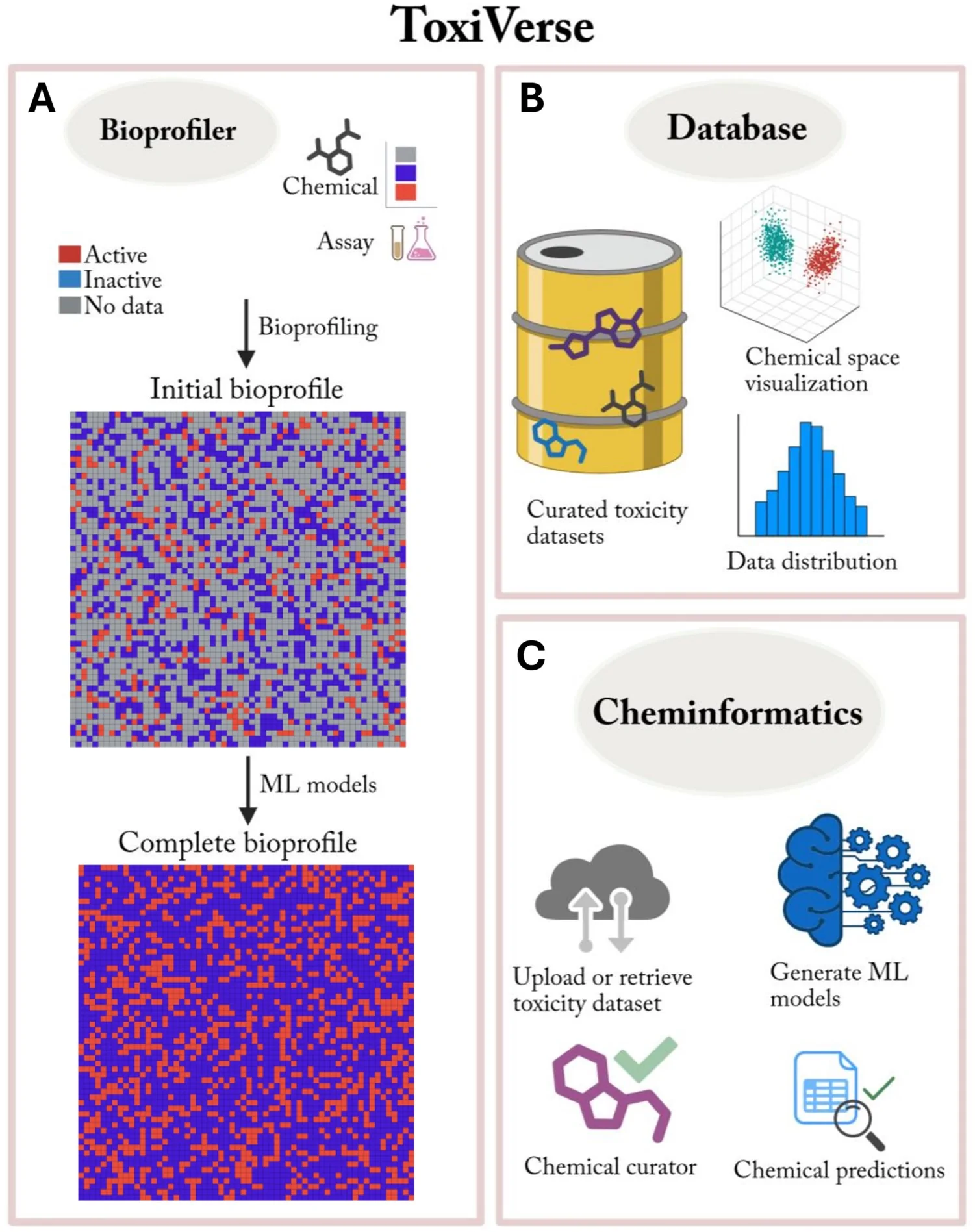
Overview of the three main modules in ToxiVerse. (A) Bioprofiler, (B) Database, and (C) Cheminformatics. The functions available within each module are shown.

ToxiVerse is a web-based informatics platform designed to provide researchers with a bioprofiling tool for target chemicals, curated chemical toxicity datasets, and computational toxicity modeling protocols within a user-friendly working environment. To improve user accessibility, the ToxiVerse tutorial is provided as a searchable web document at https://toxiverse.com/tutorial. The tutorial is organized according to the three main modules of ToxiVerse and provides step-by-step guidance for using each module. The three modules of ToxiVerse and their functions are shown in Fig. 1.

The Bioprofiler module constructs initial bioprofile for chemicals by leveraging bioassay data from PubChem and provides complete bioprofile after data gaps are filled with ML models. It allows users to input chemicals and generates a chemical-bioactivity matrix (i.e. an initial bioprofile), which can be used to identify key assays relevant to the chemicals of interest (i.e. assays that distinguish between Actives and Inactives). ML models built from chemicals in selected assays are then used to impute prediction values for input chemicals lacking experimental data in these assays. The Database module hosts a curated collection of approximately 50 000 chemicals, mainly covering toxicological datasets along with detailed data information. The module includes functions to download selected datasets and access PubChem bioassays relevant to specific toxicological endpoints. The Cheminformatics module provides options for dataset upload or retrieval from PubChem, dataset curation, visualization, and QSAR modeling and prediction. Users can upload their own datasets, use the ToxiVerse datasets provided, or import data directly from PubChem for modeling and prediction purposes.

### 3.2 Bioprofiler

The Bioprofiler module enriches the bioactivity of target chemicals by integrating PubChem assay outcomes with model-predictions for untested assays. This approach provides bioactivity-based chemical descriptors that embed compounds in a biologically informed feature space that extends beyond chemical structure alone. High-throughput screening (HTS) assays in PubChem form the basis of this approach by revealing how chemicals interact with molecular targets and indicating potential toxicity pathways (Kim *et al.* 2025). To create biologically enriched data for target chemicals, the Bioprofiler first constructs a chemical-bioactivity matrix (initial bioprofile). MI scoring is then applied to select the most informative assays for downstream modeling. Assay-specific RF models are used to impute missing outcomes, producing a more complete bioprofile for each compound. This gap-filling strategy enables chemical read-across and ensures that target compounds are represented with comprehensive bioactivity profiles. A key advantage of this activity-based profiling is the ability to achieve mechanistic related biological data for target chemicals. Compounds with little structural resemblance may nevertheless display similar activity across assays, reflecting shared mechanisms of action or phenotypic effects. By combining observed and predicted assay outcomes, the resulting hybrid descriptors integrate structural and biological information. This enriched potential training data can improve comprehensive toxicity modeling compared with structure-only descriptors. Overall, the Bioprofiler module transforms sparse HTS data into comprehensive bioactivity descriptors for target compounds. This maximizes the value of public big data and supports computational toxicology, risk assessment, and read-across applications. The integration of chemical-bioactivity profiling with statistical analysis and data gap filling has been validated across multiple toxicity prediction studies, demonstrating its reliability and effectiveness (Jia *et al.* 2022, Chung *et al.* 2023, Chung *et al.* 2024, Daood *et al.* 2024).

#### 3.2.1 PubChem bioassay data

To facilitate efficient retrieval of relevant bioactivity data and other useful information (e.g. chemical identifiers) for user-input chemicals, an SQLite database was generated from PubChem data containing chemicals and their assay outcomes. Each database release is provided with an explicit version tag based on the PubChem download date, for example, Complete Bioprofile_vMay19th2026. The database is updated every six months to remain consistent with current PubChem releases. The resulting database integrates assay bioactivities with standardized unique chemical identifiers and structural representations, enabling rapid querying without repeated network inquiries to PubChem servers, which would otherwise require significant time. The current SQLite database includes CIDs, AIDs, activity outcomes and InChI strings collected from PubChem.

#### 3.2.2 Bioprofiling

The core functions of the Bioprofiler module are shown in Fig. 1A. Bioprofiler module supports automatic bioassay data retrieval from PubChem database for input chemicals. Bioactivity data are collected from PubChem assays that have tested the input chemicals. The initial bioprofile contains AIDs (assay identifiers), CIDs (chemical identifiers), and activity outcomes. Activity outcomes are encoded as 1 for Actives, −1 for Inactives, and 0 for Inconclusive/Unspecified/No data. When different results exist for the same CID-AID pair, the active value is retained.

**Figure 2:**
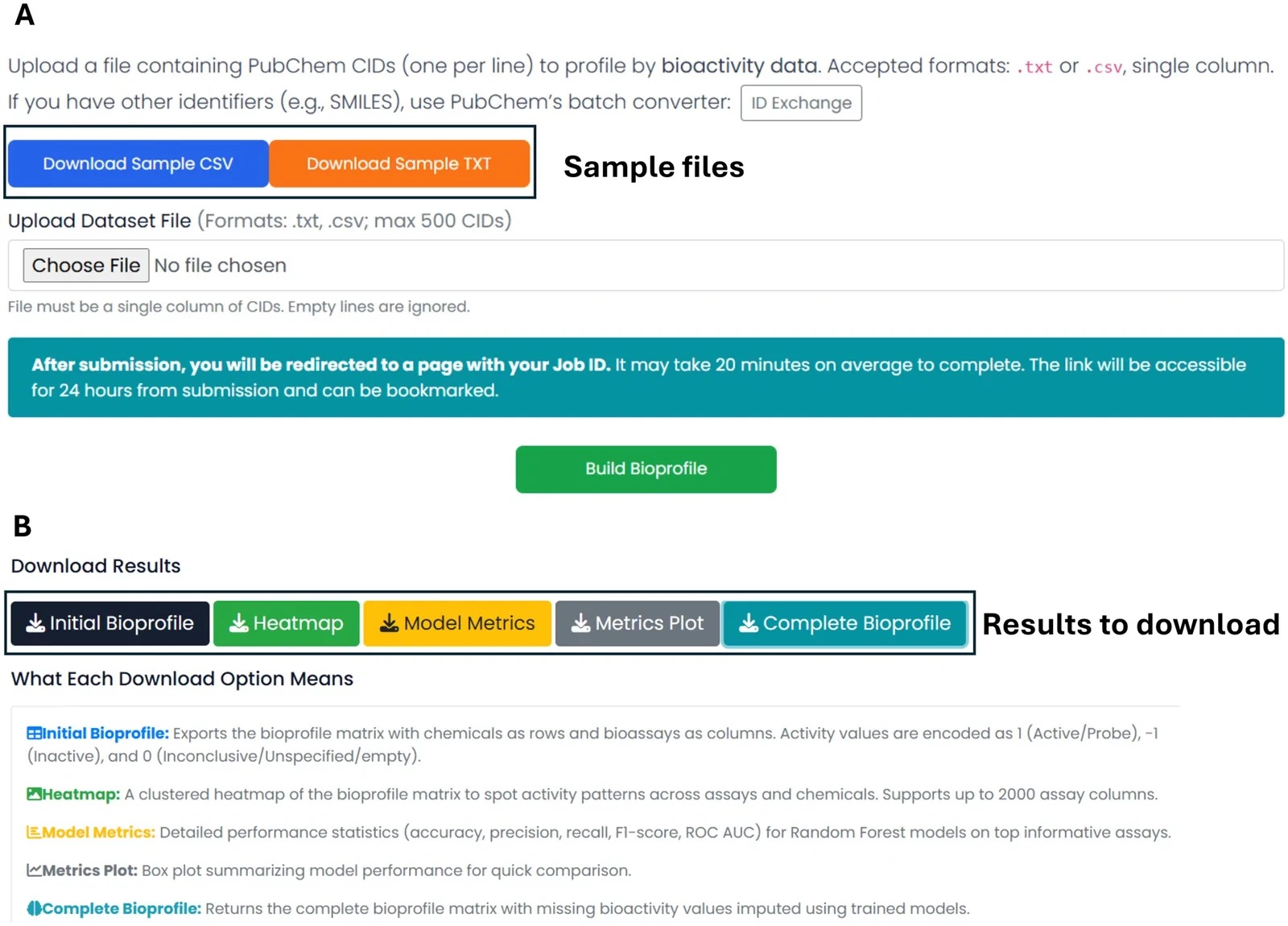
The Bioprofiler interface. It shows (A) input options for file upload and (B) options for downloading results, along with result interpretation.

**Figure 3:**
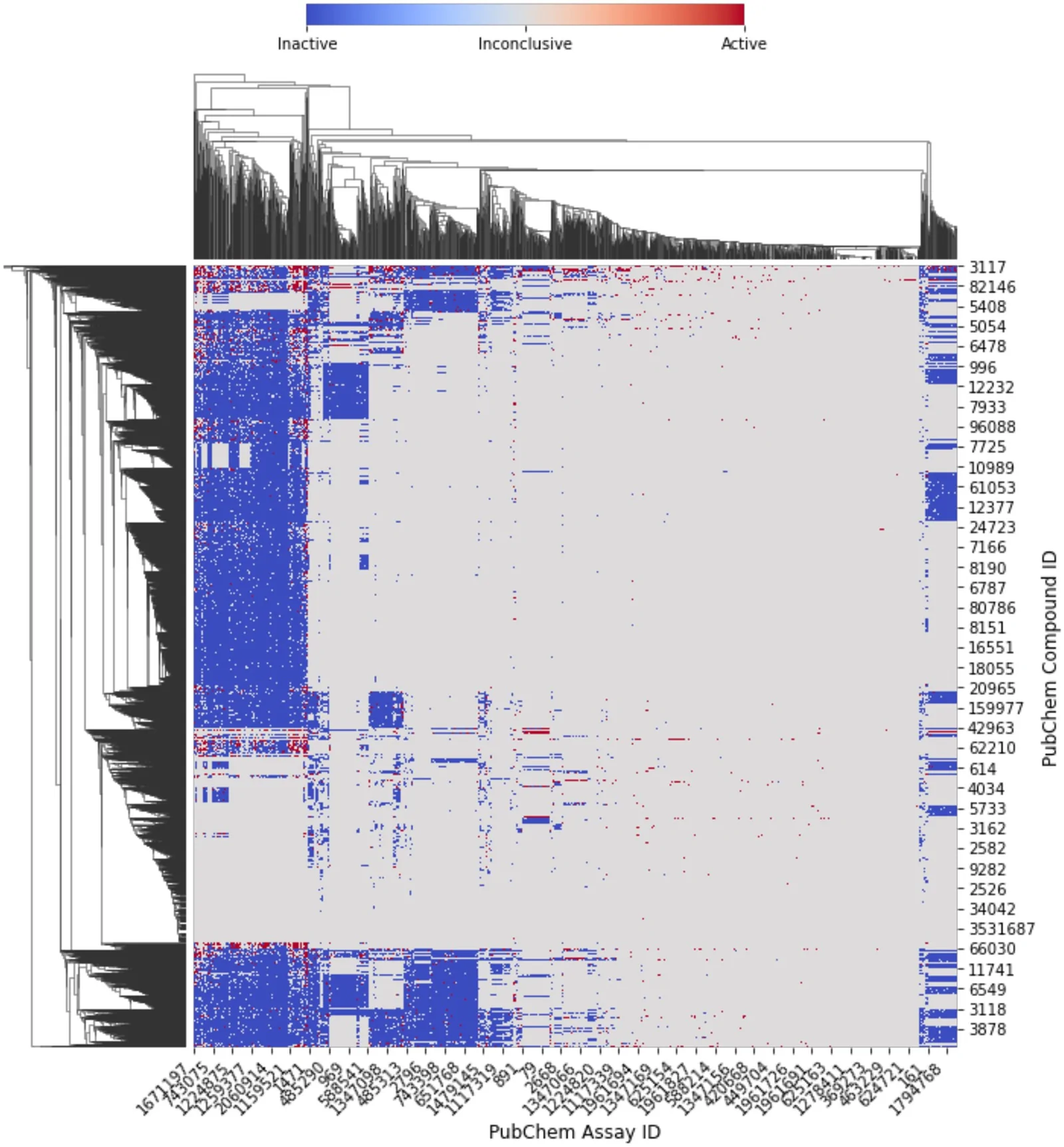
Heatmap of the bioprofile for an estrogen receptor dataset. Red indicates active (1), blue indicates inactive (−1), and grey indicates inconclusive 0.

Figure 2 displays the Bioprofiler interface for file upload and results download. The initial bioprofile is a high-dimensional matrix with chemicals as rows and assays as columns. Only assays with at least one active compound are retained. Users can download this matrix via the *Initial Bioprofile* option and a heatmap representation via the *Heatmap* option. A clustered heatmap generated from 1622 PubChem assays for an estrogen receptor dataset of 6543 compounds (Ciallella *et al.* 2021) is shown in Fig. 3.

MI scores are computed to rank assays for their usefulness in data analysis and modeling. A high MI score indicates an assay can be used to explain potential mechanisms of target endpoint, making it highly informative for modeling. Since target chemicals have missing experimental data against many PubChem assays, data gaps in the initial bioprofile are filled using chemicals originally tested in these assays. For each selected assay, up to 500 actives and 500 inactives are randomly selected from the chemicals being tested, and only assays with at least 100 chemicals in both classes are retained to ensure resulting model predictivity. Binary RF models are trained using ECFP6 fingerprints generated in RDKit. Model evaluation file is available via the *Model Metrics* option, and plot can be obtained via the *Metrics Plot* option. The trained models are then used to predict assay outcomes for target chemicals lacking experimental data (previously encoded as 0), producing a complete bioprofile downloadable via the *Complete Bioprofile* option. The results can be accessed for up to 24 hours from job submission via unique job link. The results of entire Bioprofiler module are presented in Fig. S1, available as supplementary data at *Bioinformatics* online. The performance metrics from the acute toxicity dataset (Wu *et al.* 2023) are shown as a box plot in Fig. S1B, available as supplementary data at *Bioinformatics* online. For input chemicals with CIDs, five downloadable outputs are available (Fig. 2B).

To evaluate the contribution of RF-imputed bioprofiles as potential descriptors for model development, we performed an ablation study across important toxicity endpoints (Hansen *et al.* 2009, Richard *et al.* 2021, Wu *et al.* 2021, Chung *et al.* 2024). For each endpoint, RF models were trained using three descriptor sets: structure-only ECFP6 fingerprints, selected bioprofile descriptors containing observed assay values only, and complete bioprofile descriptors containing observed plus RF-imputed assay values. The complete bioprofile descriptors are the direct outputs from the Bioprofiler module of ToxiVerse and selected bioprofile descriptors were prepared using ‘Initial bioprofile’ result from Bioprofiler module based on assay selection function. The complete bioprofile models generally showed improved or comparable performance relative to the structure-only models, while the selected bioprofile models did not perform as well as the other two models, supporting the necessity of including imputed bioprofile values. The input datasets for Bioprofile and performance metrics for all models are provided as Supplementary File S1 and Fig. S2, available as supplementary data at *Bioinformatics* online.

### 3.3 Database

**Figure 4:**
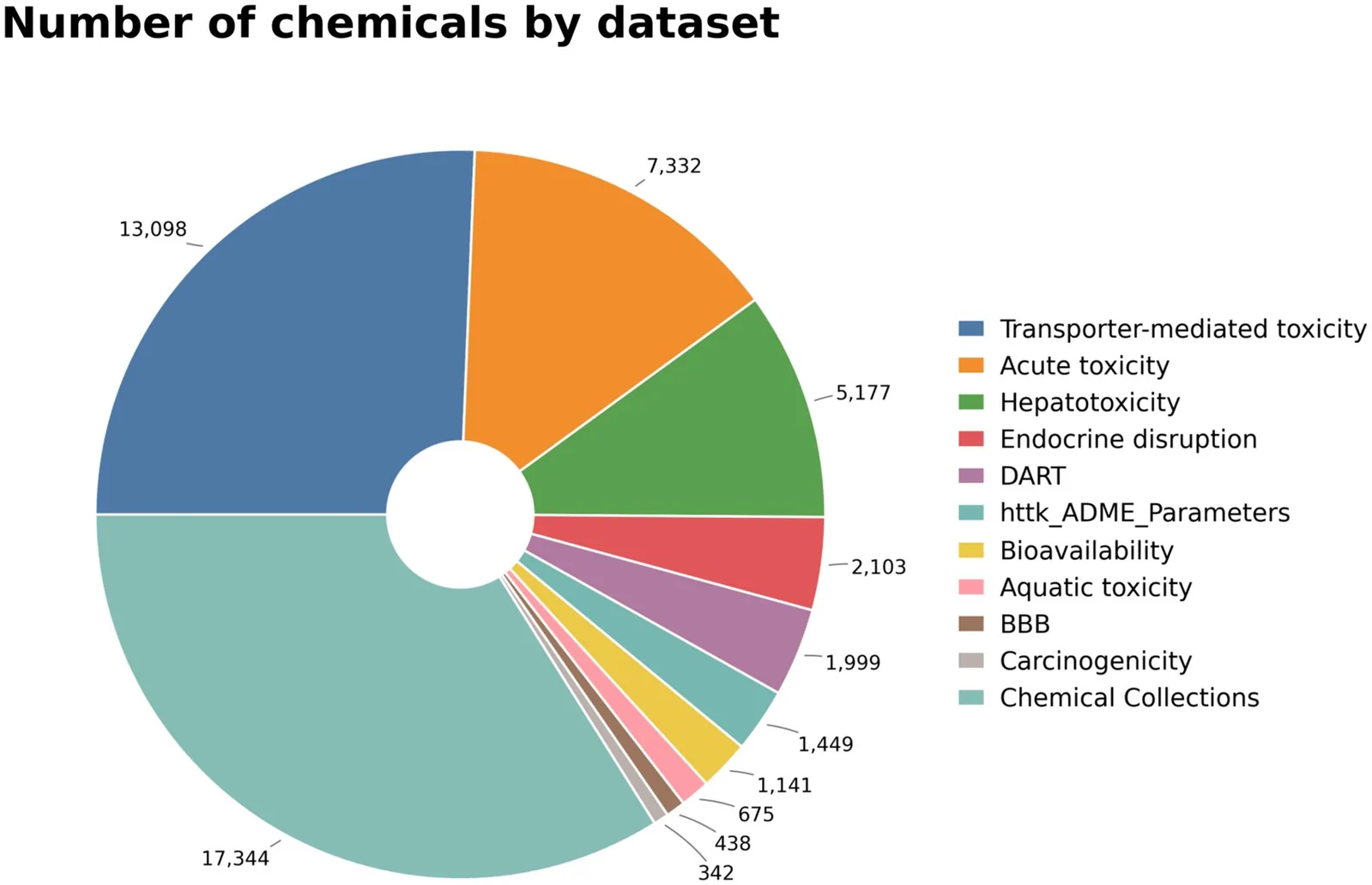
Chemical counts in the ToxiVerse Database. Number of chemicals in each dataset by category.

The *Database* module provides curated datasets that are standardized, user-friendly to access, and ready for modeling. High-quality curated datasets are critical for advancing computational toxicology (Wang *et al.* 2024, Seal *et al.* 2025). The database currently contains approximately 50 000 chemicals drawn primarily from our previous studies (Klopman and Stuart 2003, Moda *et al.* 2007, Zhu *et al.* 2009, Sedykh *et al.* 2013, Kim *et al.* 2014, Wang *et al.* 2015, Mulliner *et al.* 2016, Zhao *et al.* 2017, Ciallella *et al.* 2021, Ciallella *et al.* 2022b, Aljarf *et al.* 2023, Chung *et al.* 2023, Knox *et al.* 2024), complemented by additional toxicity data from public resources. These datasets cover toxicity annotation data across more than 50 endpoints, including widely studied toxicity areas such as acute toxicity, hepatotoxicity and endocrine disruption. ToxiVerse also provides a large collection of various types of chemicals, such as Cosmetics, High Production Volume chemicals, Natural Products, and Pesticides, for screening purposes (Fig. 4). Table S1, available as supplementary data at *Bioinformatics* online lists each source dataset, endpoint, data type, activity threshold where applicable, original reference or data source, total number of chemicals, and classification counts. Each dataset consists only of unique chemicals linked to well-known identifiers (SMILES, and PubChem CID) along with ToxiVerse ID, making them suitable for use in cheminformatics modeling pipelines.

Data accessibility of this platform is easy and user friendly. Users can interactively explore chemical space, view endpoint data distributions, and download curated CSV files with a single click. By consolidating multiple toxicity resources into a single harmonized framework, ToxiVerse overcomes common challenges of data availability and usability, ultimately promoting broader adoption of computational approaches in toxicology.

**Figure 5:**
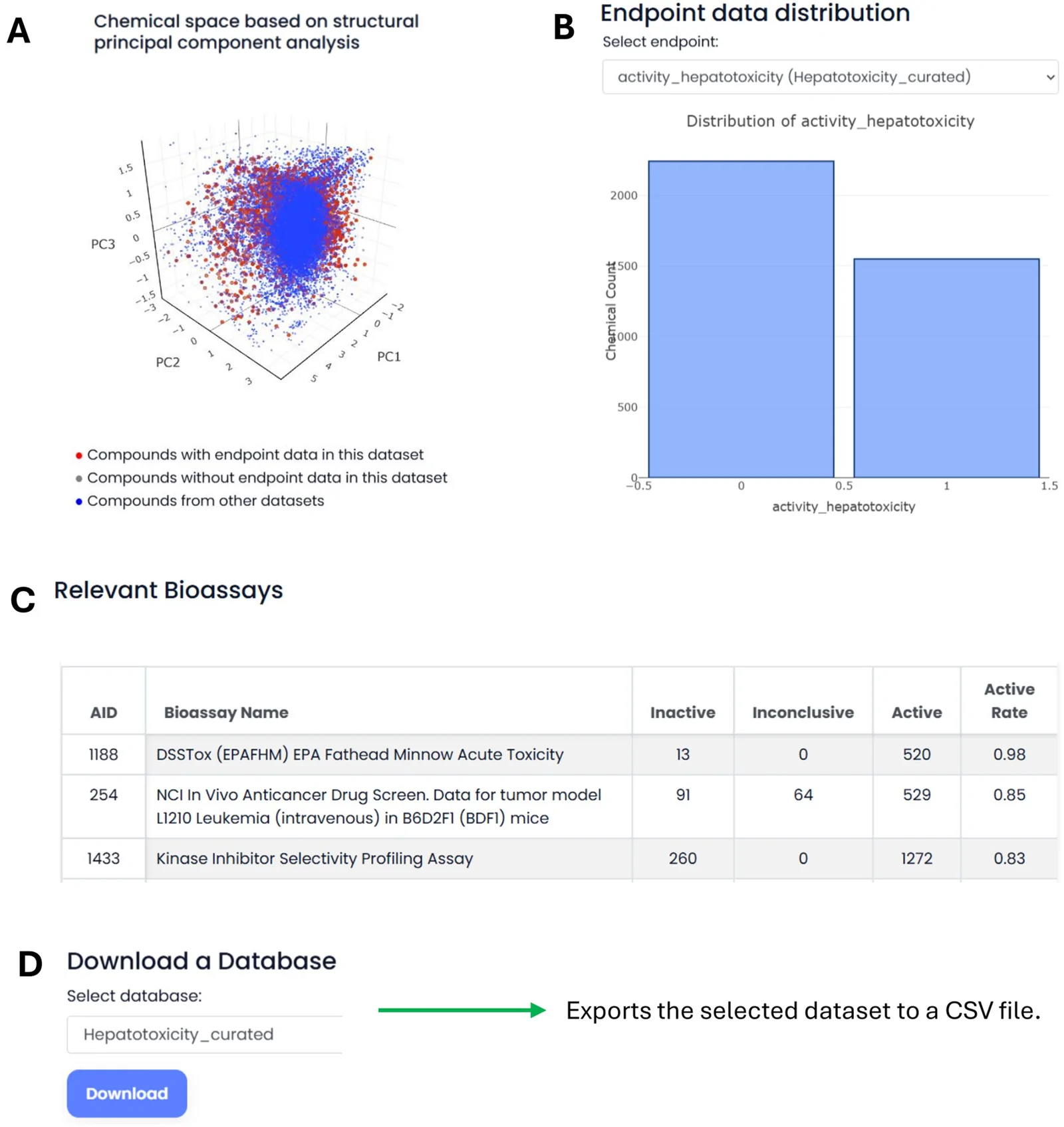
Overview of the Database module functions. (A) Chemical space visualization. (B) Endpoint data distribution. (C) Relevant bioassays for a selected endpoint. (D) Menu for downloading a selected dataset.

When a toxicity endpoint in a dataset is selected, users have three integrated views of the relevant data (Fig. 5): a PCA plot that shows the chemical space distribution of the chemicals in the dataset, a histogram illustrating the distribution of activity/toxicity values across chemicals, and a list of the 500 PubChem assays most relevant to the endpoint, identified using the Bayesian scoring approach (2 and 3).

### 3.4 Cheminformatics

**Figure 6:**
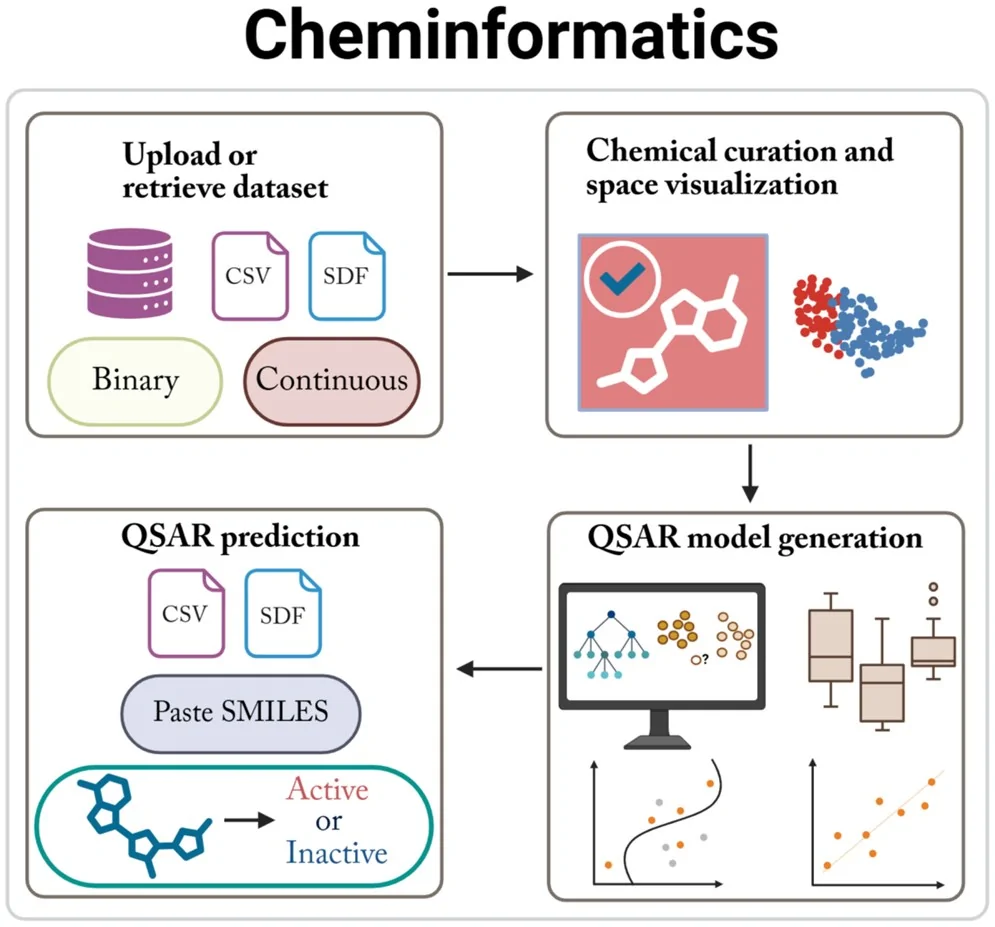
Functionalities of the Cheminformatics module. The available input options, along with their corresponding functions, are shown.

**Figure 7:**
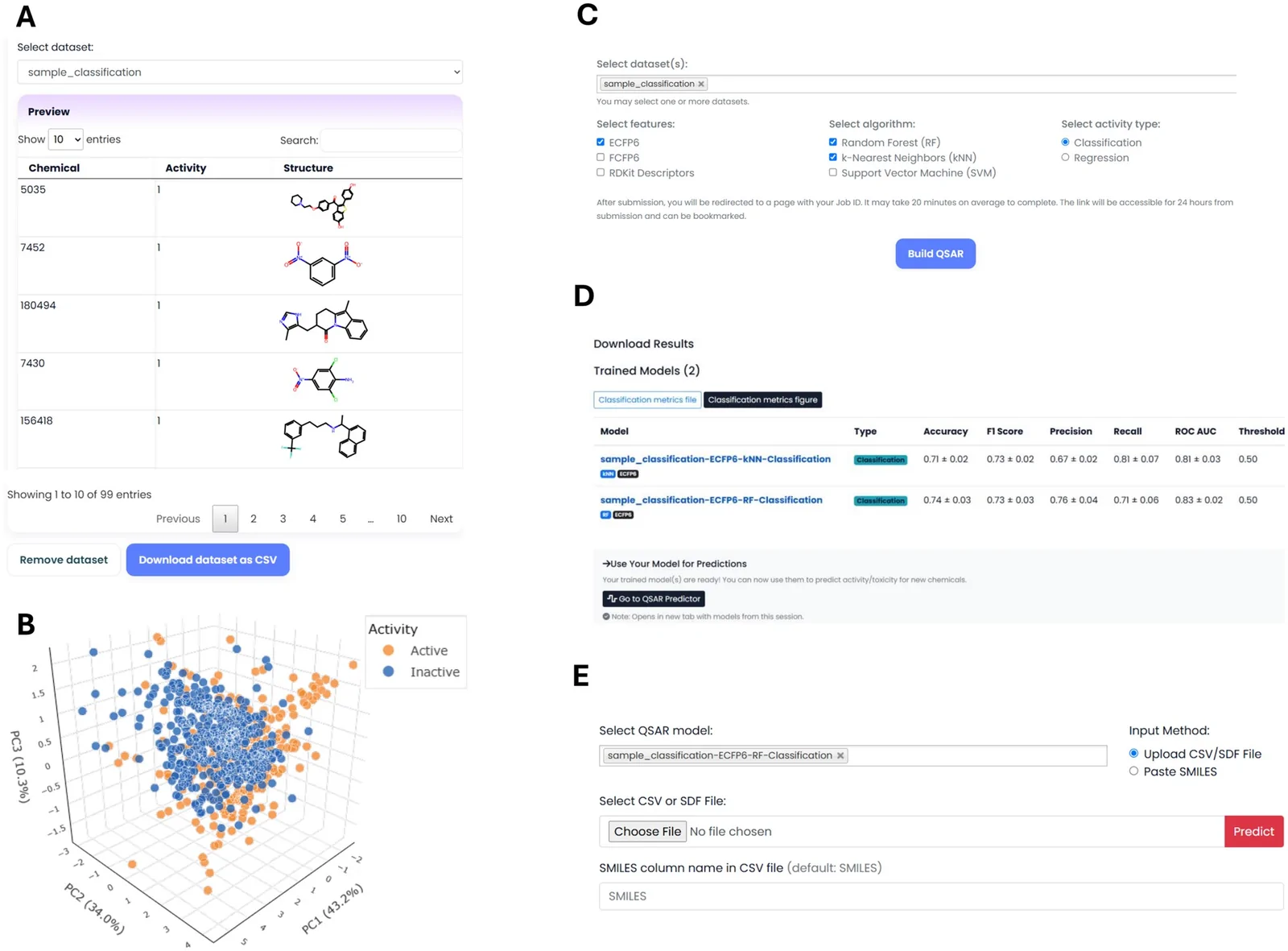
Example of main functions in the Cheminformatics module using a sample classification dataset. (A) Display of the uploaded dataset in tabular format. (B) Chemical space visualization of the dataset. (C) Selection of an uploaded dataset for QSAR model generation. (D) Display of model metrics in tabular format and the link to access the generated models. (E) Prediction of new chemicals using a selected model.

The Cheminformatics module provides a modeling workflow of dataset upload, chemical curation, visualization, and QSAR modeling (Fig. 6). The module allows users to efficiently generate machine learning models, compare their performance, and make predictions. ToxiVerse provides sample datasets for users to perform the functions in the modeling and prediction workflow (Fig. 7). This module enables researchers without programming expertise to carry out end-to-end QSAR modeling using ToxiVerse tools.

#### 3.4.1 Dataset upload and retrieval

It enables users to upload datasets in SDF or CSV formats for computational modeling. The interface accepts data that includes user-defined column names for compound identifiers, SMILES, and activity values, along with the dataset type (binary or continuous). The uploaded dataset displayed in the interface can be downloaded as a CSV file using the *Download Dataset as CSV* option or removed using the *Remove Dataset* option (Fig. 7A). Datasets can also be imported from PubChem using associated AIDs. The chemical-bioactivity records are retrieved initially for a given AID and activity outcomes are standardized by discarding *inconclusive* or *unspecified* results. If duplicate compound entries are present, the entry with a defined activity value is retained and duplicates are removed. The cleaned dataset is merged with InChI representations.

#### 3.4.2 Structure curation and chemical space visualization

Chemical structure curation uses the ChEMBL Structure Pipeline with RDKit. This process identifies and corrects problematic structures, ensuring standardized, high-quality structures for modeling. This function also allows users to choose how duplicates should be handled, by retaining the highest or lowest activity, averaging activity values, or removing duplicates entirely. Users can choose to either overwrite the original dataset with the curated version or create a new dataset, in which case “_curated” will be added to the original dataset name. In the latter case, both the uploaded and curated datasets remain accessible. For chemical space visualization, PCA is performed on a set of molecular descriptors (calculated via RDKit). The resulting 3D plot, which uses the top three principal components as the coordinates, color-codes compounds by their activity value, allowing users to visually explore structural patterns or clusters in their dataset (Fig. 7B).

#### 3.4.3 QSAR model generation

The QSAR builder supports building ML models for toxicity datasets selected by users (Fig. 7C and D). Molecular descriptors or fingerprints (Morgan/ECFP, FCFP, and RDKit descriptors) are computed and normalized when necessary. Classification and regression modeling using RF, SVM, and *k*-NN algorithms can be performed. Models are tuned using grid search with five-fold cross-validation. For classification, a default probability threshold of 0.5 is used to assign binary labels for balanced endpoints, whereas imbalanced endpoints use a threshold estimated from cross-validated out-of-fold predictions. The best-performing model is saved for future predictions. Performance metrics are accuracy, AUC, F1-score, precision, recall and ROC AUC for classification. Regression models are evaluated using *R*², Mean Squared Error (MSE), and Mean Absolute Percentage Error (MAPE). For example, we modeled a toxicity dataset (Supplementary File S2, available as supplementary data at *Bioinformatics* online) of 888 compounds (444 actives, 444 inactives) (Ciallella *et al.* 2022a) from a PubChem estrogen receptor antagonist assay (AID 1259248). Nine classification models (combinations of ECFP6, FCFP6, RDKit descriptors with kNN, RF, SVM) were built and evaluated via five-fold cross-validation. We have also generated classification models for an imbalanced endpoint using the neurotoxicity Tox21 dataset AID1347395, which contains 1527 chemicals, including 382 actives and 1145 inactives. The results are shown in Fig. S3, available as supplementary data at *Bioinformatics* online. The QSAR builder option allows users to download performance metrics plot for their models, along with the corresponding performance scores in a CSV file (Fig. 7D). The results can be accessed for up to 24 hours from job submission via unique job link.

To show the head-to-head comparison of ToxiVerse model development to other available web-based toxicity modeling tools, we compared the prediction results of ToxiVerse-built models with vNN-built models for important toxicity endpoints. The vNN method is a similarity-based QSAR approach used to predict ADMET properties. It defines an applicability domain using a Tanimoto-distance threshold and assigns greater weight to closer structural neighbors through a smoothing factor. First, classification models of five toxicity endpoints, such as hERG-related cardiotoxicity (Wu *et al.* 2021), hepatotoxicity (Chung *et al.* 2024), AMES mutagenicity (Hansen *et al.* 2009), neurotoxicity (Richard *et al.* 2021), and endocrine disruption (Estrogen receptor alpha) (Richard *et al.* 2021), were generated in ToxiVerse using RF algorithms and ECFP6 fingerprints and the vNN web server through the ‘Run Custom Model’ option (Schyman *et al.* 2017). For vNN model building, a Tanimoto distance of 0.7 and a smoothing factor of 0.2 were used, based on most values used in vNN web server. Predictions were generated using the same external validation sets, which were not included in the corresponding training datasets. The prediction performance of ToxiVerse was comparable to that of vNN, and the training datasets, external validation datasets, and prediction results are provided as Supplementary Files S2–S12, available as supplementary data at *Bioinformatics* online.

#### 3.4.4 QSAR prediction

The QSAR prediction interface allows users to predict activities/toxicities for chemicals using trained models (Fig. 7E). Chemicals to be predicted can be provided via file upload (CSV or SDF) or by direct SMILES input. Invalid structures are automatically removed. For CSV uploads, the system automatically detects a column named “SMILES,” or users can manually specify the SMILES column name. A “Paste Sample SMILES” feature is provided to help users quickly make predictions without uploading files.

To improve the reliability of QSAR predictions, an applicability domain (AD) assessment was implemented within the ToxiVerse QSAR Predictor. The AD is calculated using nearest-neighbor Tanimoto similarity between the query molecule and compounds in the corresponding model training set. For each model, a model-specific AD threshold is defined as the fifth percentile of leave-one-out nearest-neighbor Tanimoto similarities among the training-set compounds. For each query SMILES, ToxiVerse reports the predicted activity label, the closest training-set compound, the maximum Tanimoto similarity, and the AD status. Query molecules with similarity equal to or higher than the model-specific AD threshold are considered within the model’s training chemical space, whereas molecules with similarity below the threshold are flagged as outside the AD. Predictions outside the AD should be considered low-confidence predictions. The AD information is shown as the last column of the prediction results table and is also included in the downloadable CSV output.

### 3.5 Distinctive features of ToxiVerse

The unique features of ToxiVerse compared with other ADMET tools are provided in Supplementary Table S2, available as supplementary data at *Bioinformatics* online. Despite the valuable contributions of other existing tools, most rely on fixed, pre-trained models for a predetermined set of endpoints, offering little to no flexibility for users to input new datasets or build customized predictive models with several chemical descriptors and ML algorithms (Seal *et al.* 2025). This rigidity restricts their utility where new data or endpoints need to be modeled and evaluated. Additionally, batch processing capabilities are often lacking. While many platforms are web-accessible, their functionalities can still pose barriers for users without programming backgrounds. ToxiVerse addresses these challenges through a fully integrated, web-based architecture that enables users to build custom QSAR models using either curated ToxiVerse datasets or their own experimental data. Its Bioprofiler module enriches chemical descriptors by leveraging high-throughput bioactivity data from PubChem and incorporates machine learning-based imputation to fill data gaps of selected assays. The platform also provides access to a rigorously curated database for various toxicity endpoints. Furthermore, ToxiVerse supports batch processing and step-by-step model building without requiring coding skills. By combining flexibility, mechanistic insight, and user accessibility in a single platform, ToxiVerse fills some of the critical usability and adaptability gaps left by earlier toxicity prediction tools.

## Supplementary Material

btag617_Supplementary_Data

## Data Availability

The ToxiVerse code is openly accessible through the GitHub repository at: https://github.com/zhu-research-group/toxiverse. Archived snapshot of the code is available on Zenodo at: https://doi.org/10.5281/zenodo.20727721.
