# Supplementary material for: ToxiVerse: chemical bioprofiling, toxicity data sharing and customizable predictive modeling
Source: Bioinformatics. 2026 Aug 22;42(9):btag617. doi: 10.1093/bioinformatics/btag617 (PMC13533561; doi:10.1093/bioinformatics/btag617)
Supplement: Supplementary file 1 [file btag617_supplementary_data.zip › ToxiVerse_Supplementary material_Final modifications_Aug12th2026.docx]

**Supplementary Information**

# ToxiVerse: Chemical Bioprofiling, Toxicity Data Sharing and Customizable Predictive Modeling

*Prasannavenkatesh Durai^1^, Daniel P. Russo^2^, Yitao Shen^1,2^, Tong Wang^1,2^, Elena Chung^1,2^, Lang Li^3^, Hao Zhu^1,2*^*

^1^Center for Biomedical Informatics and Genomics, Tulane University School of Medicine,

New Orleans, LA 70112, United States.

^2^Department of Chemistry and Biochemistry, Rowan University, Glassboro, NJ 08028, United States

^3^Department of Biomedical Informatics, College of Medicine, The Ohio State University, Columbus, OH 43210, USA.

**Supplementary Figure S1.** The Bioprofiler functions overview: generating five distinct outputs, each available for download with sample outputs provided. (A) Heatmap representation of initial bioprofile. (B) Box plot of performance metrics for models built on the selected assays. (C) The initial bioprofile shown as chemical-bioactivity matrix with a tabular format of compound activity outcomes (1 = active, -1 = inactive and 0 = inconclusive). (D) The performance metrics file containing evaluation results for the models based on selected assays. (E) A complete bioprofile with filled data gaps. A represents Assay and C represents chemical in the figure.

**Supplementary Figure S2:** Performance comparison of structure-only, selected bioprofile, and complete bioprofile models.

**Supplementary Figure S3:** Performance metrics of the ToxiVerse models for balanced (Estrogen receptor alpha) and imbalanced (Neurotoxicity) endpoints.

| **Dataset Name** | **Total # of Compounds** | **Data Type** | **Dataset Source** | **Dataset Description** |
| --- | --- | --- | --- | --- |
| Acute toxicity | 7332 | -log10 mol/kg-bw | [Zhu et al.](https://pubmed.ncbi.nlm.nih.gov/19672406/) (DOI: 10.1289/ehp.0800471) | Publicly available rat oral acute toxicity data with LD₅₀ values. |
| Aquatic toxicity | 675 | -log10 of Conc. (µmol/L) | [Klopman et al.](https://academic.oup.com/etc/article/19/2/441/7831290) (https://doi.org/10.1002/etc.5620190225) | Standardized 96-hour LC₅₀ toxicity data for *Pimephales promelas* (fathead minnow) using publicly available toxicology resources. |
| BBB (Blood Brain Barrier) | 438 | logBB | [Wang et al.](https://doi.org/10.1007/s11095-015-1687-1) (https://doi.org/10.1007/s11095-015-1687-1) | Compounds with experimental logBB values were compiled from various public resources and curated using ChemAxon and CASE Ultra tools. |
| Bioavailability | 1141 | oral bioavailability (%F) | [Kim et al.](https://link.springer.com/article/10.1007/s11095-013-1222-1) (https://doi.org/10.1007/s11095-013-1222-1) [Moda et al.](https://www.sciencedirect.com/science/article/pii/S096808960700764X?via%3Dihub) (DOI: 10.1016/j.bmc.2007.08.060) | Compiled from public databases and literature sources. Chemical structures were standardized, and reported %F values were harmonized to resolve discrepancies among sources. |
| Carcinogenicity | 342 | Binary, 0=Non-Carcinogen; 1=Carcinogen | [Chung et al.](https://pubs.acs.org/doi/10.1021/acs.est.3c00648) (https://doi.org/10.1021/acs.est.3c00648) | 342 unique organic compounds (59 carcinogenic and 283 non-carcinogenic) from the EPA’s IRIS database, labeled as carcinogenic or noncarcinogenic based on oral slope factor (OSF), a quantitative measure for oral cancer risk. |
| Cosmetics | 4129 | Activity_Cosmetics (All chemicals fall under the cosmetic category) | [Chung et al.](https://pubs.acs.org/doi/10.1021/acs.est.3c00648) (https://doi.org/10.1021/acs.est.3c00648) | Collected from COSMOS Cosmetics Inventory knowledge base. |
| DART (Developmental and Reproductive Toxicity) | 1999 | Binary Labels (1:Safe; 0: Teratogen); Activity_DART (1:Toxic; 0:Non-toxic) | [Ciallella et al.](https://pubs.acs.org/doi/10.1021/acs.est.2c01040) (https://doi.org/10.1021/acs.est.2c01040) [Aljarf et al.](https://pubs.acs.org/doi/10.1021/acs.jcim.2c00824) (https://doi.org/10.1021/acs.jcim.2c00824) | Collected from public databases and literature sources, including ECHA, ToxRefDB, and the Procter & Gamble dataset, using mammalian prenatal developmental toxicity studies involving oral or inhalation exposure. EmbryoTox dataset was curated from Australian Therapeutic Goods Administration (TGA) pregnancy classification data. |
| Drugbank | 8055 | Activity_Drugbank (All chemicals fall under the drug category) | [Chung et al.](https://pubs.acs.org/doi/10.1021/acs.est.3c00648) (https://doi.org/10.1021/acs.est.3c00648) | Collected from DrugBank. |
| Endocrine disruption | 2103 | Agonist, Antagonist, Binding, and Uterotrophic class | [Ciallella et al.](https://www.laboratoryinvestigation.org/article/S0023-6837(22)00653-5/fulltext) (DOI: 10.1038/s41374-020-00477-2) | The dataset was compiled from CERAPP, which provides in vitro estrogen receptor agonism, antagonism, and binding data, and from EADB, which contains in vivo rodent uterotrophic assay data. |
| Hepatotoxicity | 5177 | Several classification endpoints for hepatotoxicity are provided at standard or dose-based thresholds. Activity is the main endpoint. Endpoints labeled H-* represent human data, and endpoints labeled PC-* represent preclinical data. The abbreviations used include: CC – clinical chemistry, HC – hepatocellular injury, HT – hepatotoxicity, HB – hepatobiliary injury, and MF – morphological findings. | [Mulliner et al.](https://pubs.acs.org/doi/10.1021/acs.chemrestox.5b00465) (https://doi.org/10.1021/acs.chemrestox.5b00465) | Compiled from published literature, DrugBank, PharmaPendium, Leadscope, and internal 14–28 day rat study data. |
| High Production Volume chemicals | 1672 | Activity_HPV (All chemicals fall under the HPV category) | [Chung et al.](https://pubs.acs.org/doi/10.1021/acs.est.3c00648) (https://doi.org/10.1021/acs.est.3c00648) | Collected from the U.S. EPA High Production Volume (HPV) Challenge Program chemical database. |
| Httk_ADME_Parameters | 1449 | Human CLint (intrinsic clearance) in µL/min/10^6 cells, Human Fu (fraction unbound), Rat CLint (intrinsic clearance) in µL/min/10^6 cells, and Rat Fu (fraction unbound) | [High-Throughput Toxicokinetics](https://cran.r-project.org/web/packages/httk/index.html) (https://cran.r-project.org/web/packages/httk/index.html) | The HTTK dataset, developed by the U.S. EPA, contains high-throughput toxicokinetic data and models covering pharmaceuticals and environmental chemicals. It includes *in vitro* measurements like plasma protein binding and hepatic clearance rates, as well as species-specific physiological data such as tissue volumes and blood flow rates. |
| Natural products | 2479 | Activity_NP (All chemicals fall under the natural product category) | [Chung et al.](https://pubs.acs.org/doi/10.1021/acs.est.3c00648) (https://doi.org/10.1021/acs.est.3c00648) | Curated from the Traditional Chinese Medicine Systems Pharmacology Database and Analysis Platform (TCMSP). |
| Pesticides | 1009 | Activity_Pesticides (All chemicals fall under the pesticide category) | [Chung et al.](https://pubs.acs.org/doi/10.1021/acs.est.3c00648) (https://doi.org/10.1021/acs.est.3c00648) | Collected from literature and public databases, including the U.S. EPA CompTox Chemistry Dashboard. |
| Transporter-mediated toxicity (Classification data) | 10,875 | **Proteins:** • P-glycoprotein (P-gp; MDR1) • Breast cancer resistance protein (BCRP) • Multidrug resistance–associated proteins (MRP1, MRP2) • Bile salt export pump (BSEP)  **Activity values:** • Substrate and Inhibitor calls • Evidence of inhibition thresholds. | [Daood et al.](https://pubs.acs.org/doi/10.1021/acs.molpharmaceut.5c01065) (https://doi.org/10.1021/acs.molpharmaceut.5c01065) [Sedykh et al.](https://link.springer.com/article/10.1007/s11095-012-0935-x) (https://doi.org/10.1007/s11095-012-0935-x) [Zhao et al.](https://pubs.acs.org/doi/10.1021/acsomega.7b00274) (https://doi.org/10.1021/acsomega.7b00274) | Consolidates curated transporter data across P-gp, BCRP, MRP1, MRP2, and BSEP. Records were standardized and manually reviewed for cited literature sources, noting details such as cell lines, substrates, substrate concentrations, and positive controls where available. Sources include large-scale ChEMBL bioactivity extractions, PubChem, and Metrabase data. High-confidence entries from the Intestinal Transporter Database are included for MDR1. Substrate/inhibitor calls are harmonized, and inhibition evidence follows study-specific thresholds (10 µM for P-gp/BCRP; 100 µM for BSEP). |
| Transporter-mediated toxicity (Regression data) | 2223 | **Proteins:** • P-gp • BCRP • MRP1  **Activity values:** • -log(IC50) or pIC50 | [ChEMBL](https://doi.org/10.1093/nar/gkad1004) (doi: 10.1093/nar/gkad1004) [Papyrus](https://doi.org/10.1186/s13321-022-00672-x) (https://doi.org/10.1186/s13321-022-00672-x) | Curated *in vitro* chemical inhibition data for P-gp, BCRP and MRP1 were collected from ChEMBL and Papyrus. Records were standardized and activities were harmonized using available pChEMBL values. When duplicate entries were present for the same chemical, one representative entry was retained using the average reported activity value. |

**Supplementary Table S1:** Curated datasets included in ToxiVerse, with compound counts, data types, sources, and brief descriptions.

| **Features** | **ToxiVerse** | **v-NN** | **ADMETlab 3.0** | **ProTox 3.0** | **admetSAR 3.0** |
| --- | --- | --- | --- | --- | --- |
| **No registration required** |  |  |  |  |  |
| **Assay-based chemical descriptors** |  |  |  |  |  |
| **User-provided toxicity data for model building** |  |  |  |  |  |
| **Multiple algorithms and descriptors for model building** |  |  |  |  |  |
| **Downloadable ADMET datasets** |  |  |  |  |  |
| **Endpoint data visualization** |  |  |  |  |  |
| **Chemical curation** |  |  |  |  |  |
| **Batch screening** |  |  |  | Limited |  |
| **Key assays related to each endpoint** |  |  |  |  |  |

**Supplementary Table S2:** The unique features of ToxiVerse compared with other ADMET prediction tools.
